# MICCA: a complete and accurate software for taxonomic profiling of metagenomic data

**DOI:** 10.1038/srep09743

**Published:** 2015-05-19

**Authors:** Davide Albanese, Paolo Fontana, Carlotta De Filippo, Duccio Cavalieri, Claudio Donati

**Affiliations:** 1 Fondazione Edmund Mach, Research and Innovation Centre, Computational Biology Department, Via E. Mach 1, 38010 - S. Michele all'Adige (TN), Italy; 2 Fondazione Edmund Mach, Research and Innovation Centre, Food Quality Nutrition & Health Department, Via E. Mach 1, 38010 - S. Michele all'Adige (TN), Italy

## Abstract

The introduction of high throughput sequencing technologies has triggered an increase of the number of studies in which the microbiota of environmental and human samples is characterized through the sequencing of selected marker genes. While experimental protocols have undergone a process of standardization that makes them accessible to a large community of scientist, standard and robust data analysis pipelines are still lacking. Here we introduce MICCA, a software pipeline for the processing of amplicon metagenomic datasets that efficiently combines quality filtering, clustering of Operational Taxonomic Units (OTUs), taxonomy assignment and phylogenetic tree inference. MICCA provides accurate results reaching a good compromise among modularity and usability. Moreover, we introduce a de-novo clustering algorithm specifically designed for the inference of Operational Taxonomic Units (OTUs). Tests on real and synthetic datasets shows that thanks to the optimized reads filtering process and to the new clustering algorithm, MICCA provides estimates of the number of OTUs and of other common ecological indices that are more accurate and robust than currently available pipelines. Analysis of public metagenomic datasets shows that the higher consistency of results improves our understanding of the structure of environmental and human associated microbial communities. MICCA is an open source project.

Microbial communities are an essential part of every ecosystem ranging from marine water to soil and to the human body. The recent advancements in High Throughput Sequencing technologies have greatly increased our understanding of the role of the microbiota in different habitats and health conditions. The structure of microbial communities is often investigated through the sequencing of selected genomic markers able to characterize the sample to a given level of taxonomic resolution. The choice of these markers usually falls on the variable regions of the 16S rDNA gene if the target microbial community is composed by bacteria, or on the internal transcribed spacer (ITS) region in the case of fungal communities.

To characterize the taxonomic structure of the samples, the sequences are organized into Operational Taxonomic Units (OTUs) at varying levels of identity (usually 97%), which represent the common working definition of bacterial species. OTU clustering is a key step for the definition of the microbial diversity and taxonomic composition of the analysed samples, and its accuracy depends not only on the clustering algorithm, but also on the pre-processing of the reads for the removal of low quality reads and of chimera sequences that might be generated by template switching during the cloning step. An aggressive approach using strict quality filtering may lead to a loss of information particularly affecting the least abundant species, while, on the other hand, clustering of low quality reads may produce spurious OTUs leading to an overestimate of the complexity of the analysed samples.

Given the complexity of next generation sequencing (NGS) datasets, a common approach of the available pipelines for OTUs identification is based on incremental algorithms like the one implemented in mothur^1^, a pipeline for targeted metagenomic data incorporating several tools for sequence filtering, OTUs definition and estimate of ecological parameters. For the OTU definition mothur exploits a greedy algorithm, that assigns sequences to OTUs according to three different criteria: in *nearest neighbour* method a sequence is inserted into an OTU if it is similar to any other sequence in that group, *furthest neighbour* criteria assigns a sequence to an OTU if it is similar to all the sequences in that group and *average neighbour* method instead averages the distances among the sequences in a group. QIIME^2^, a recent workflow for metagenomic data analysis, integrates several tools in a single pipeline to analyse sequencing data including also the possibility to display the results in a graphical form. To cluster sequences into OTUs, QIIME uses UCLUST^3^, a heuristic recursive algorithm that first identifies the centroid of the OTU based on its abundance and then includes in the OTU all sequences having sequence identity above a prefixed threshold with the centroid. A recently introduced pipeline, UPARSE^4^, implements a slightly different approach than mothur and QIIME: after cleaning, quality filtering and dereplication, the input sequences are ordered according to their abundances considering that high abundance reads are more likely to be correct and then are suitable to use as cluster seeds. Given a sequence, a maximum parsimony model is found using UPARSE-REF and the read can be assigned to a known OTU, marked as chimeric (and therefore discarded) or become a new centroid. Compared to the other two tools UPARSE has been shown to be more accurate in OTU reconstruction^4^.

Here we present MICCA, a software pipeline for the processing of targeted metagenomic datasets that efficiently combines filtering process, OTU clustering and taxonomic assignment. Besides reaching a good compromise among modularity, usability and efficiency, MICCA includes OTUCLUST (Supplementary Figure S1), a new de novo OTUs clustering algorithm that improves the performances of available software. Using synthetic datasets we show that thanks to the optimized reads filtering process and to the performances of OTUCLUST, MICCA can reconstruct the structure of microbial community with higher accuracy and efficiency than currently available pipelines. Analysis of public metagenomic datasets demonstrates that the noise reduction accomplished by MICCA improves our understanding of microbial communities both in environmental and human associated samples.

## Results

MICCA is a software pipeline for the analysis of targeted metagenomic data, which includes tools for quality, chimera filtering and OTU clustering. In addition, we have also included modules for taxonomic classification, multiple alignment and phylogenetic tree inference (Table 1). MICCA can be used with any kind of amplicon reads obtained by different sequencers such as Roche 454 or Illumina. In order to assess the performances of our pipeline, we have generated and analysed three synthetic metagenomic datasets (16S-10, ITS-10 and 16S-R) of known structure and composition using MICCA, and compared the results to other popular analysis pipelines The results were analysed in term of number of reads passing the quality filtering, percentage of identified chimeras, number of OTUs and stability of the main diversity indices. To comparatively test the robustness of MICCA and other pipelines to different choices of the amplified region, we have analysed datasets derived from the Human Microbiome Project (HMP)^5^, for which the V1-V3, V3-V5 and V6-V9 variable regions of the 16S rDNA gene were amplified and sequenced.

### Quality filtering and chimera identification

To investigate the ability of MICCA to filter low quality reads and identify chimeric sequences, we have analysed the 16S-10 and ITS-10 datasets generated using a realistic error model for 454 reads and including 20% of chimera sequences. The results in term of number of reads passing the filtering and number of identified chimeras are reported in Table 2. While the number of reads discarded by MICCA and QIIME are very similar, UPARSE tends to discard a higher number of reads, keeping only 42.3% and 66.8% of the reads on the 16S-10 and ITS-10 datasets, respectively. This behaviour is due to the stringent quality filtering approach used by UPARSE^4^, which is based on truncating the reads at the first base position which has a quality score less than a specified threshold. Combined with the requirement of a minimum read length, this behaviour causes the loss of a large fraction of the sequence data and has potentially a negative impact on the ability to estimate the frequency of low-abundant species.

We have also measured the number of chimeric reads that are still present in the processed datasets from the three pipelines. The results are shown in Table 2. While the performances obtained by MICCA and QIIME are very close, UPARSE obtained the worst results in terms of number of chimeric reads retained on both 16S-10 and ITS-10 datasets (15.6% and 15.5% respectively).

### OTUs identification

**Figure 1:**
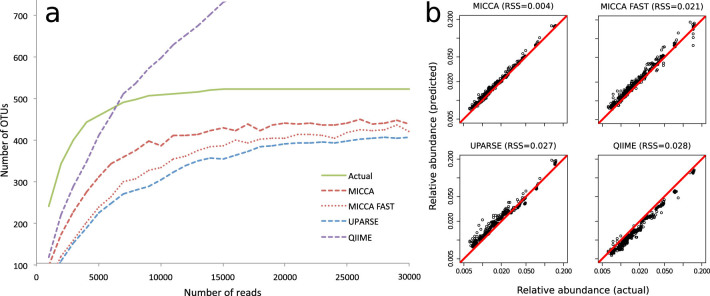
16S-R dataset: Evaluation of MICCA pipeline performance compared with UPARSE and QIIME. MICCA was also tested in fast variant (MICCA-FAST). In (a) the rarefaction curves are plotted. The continuous green lines represent the actual values. In (b) the relative abundances of the top 20 ranked OTUs compared to the actual values. RSS: Residual Sum of Squares.

To measure the ability of MICCA to correctly estimate the number of OTUs in metagenomic datasets, we have generated 16S-R, a large synthetic sequence dataset including 500 different OTUs and 100000 sequence reads. The sample was rarefied to a fixed depth of increasing size at steps of 1000 reads, obtaining 30 samples containing from 1000 to 30000 sequences and from 231 to 500 OTUs. By analysing subsamples of growing size, we expect the estimated number of OTUs to approach the actual number of species in the original dataset. Larger systematic deviations from this number are indication of decreasing performance of the software. In Figure 1a (see also Supplementary Table S1) we show the number of OTUs estimated by MICCA and MICCA-FAST, with the latter implementing an exact string matching dereplication algorithm (see OTUCLUST algorithm), as a function of the size of the rarefied sample. The green solid lines are the true number of OTUs in the subsamples, while the dashed lines are the estimates obtained by MICCA, MICCA-FAST, QIIME and UPARSE. The data demonstrates that while QIIME overestimates the complexity of the sample with no sign of levelling of the number of OTUs for increasing size of the sequence sample, UPARSE is more conservative, converging to a number of estimated OTUs that is approximately 64% on average of the real number. The performances of mothur were qualitatively similar to QIIME (Supplementary Figure S2) not reaching a plateau in the rarefaction curve and overestimating the number of OTUs^4^. Amongst the tested software suites, MICCA and MICCA-FAST achieve the best performances, converging to a number of estimated OTUs that are on average approximately 77% and 68% of the real number, respectively. In Supplementary Table S2 we also report the Shannon diversity index, a popular ecological measure used to estimate the complexity of microbial populations from metagenomic data, to highlight the effects of the more accurate estimation of the OTUs. In the case of UPARSE, the lower number of reads passing the quality filter could contribute to the lower number of estimated OTUs. To assess this point, we have repeated the analysis skipping the filtering step for MICCA and UPARSE. Even in this case, the number of OTUs predicted by MICCA is much closer to the real number (see Supplementary Figure S3), demonstrating that the superior performances of MICCA are due to the efficient clustering algorithm.

Besides estimating the biological complexity of a sample, metagenomic data provide a picture of the taxonomic structure of microbial communities. To understand the performances of the different pipelines, in Figure 1b we plot the relative abundances of the top 20 most abundant OTUs in the synthetic dataset estimated by MICCA, MICCA-Fast, QIIME and UPARSE against the effective abundances (see also Supplementary Figure S4 for mothur). Amongst the tested pipelines, MICCA shows the smallest deviation form the real relative abundances, with a value of the Residual Sum of Squares (RSS) of 0.004, to be compared with 0.027 and 0.028 for QIIME and UPARSE, respectively (0.008 for mothur). Thus, compared to other software, MICCA provides a more realistic estimate of both the number of distinct OTUs present in the sample and of the relative frequency of the most abundant samples.

**Figure 2:**
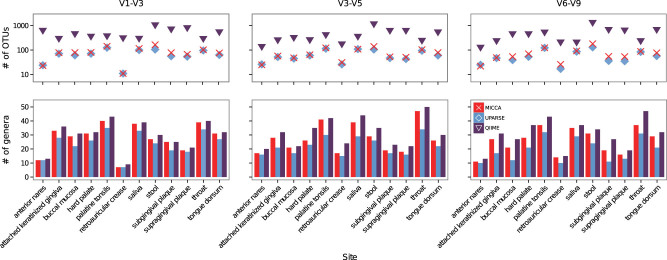
Number of OTUs (upper panels) and of distinct genera (lower panels) obtained using MICCA, QIIME and UPARSE for three choices of the of the 16S variable region, namely V1-V3, V3-V5 and V6-V9. Samples were taken from the HMP, selecting those for which data for the three regions were available.

In order to quantify to what extent the improved estimate of the OTUs given by MICCA translates into the identification of novel taxa, we have analysed samples of variable biological complexity from the Human Microbiome Project^5^ and determined the distinct bacterial genera identified by the various pipelines. To take into account possible biases introduced by the choice of the amplified region, we have selected datasets where the V1-V3, V3-V5 and V6-V9 hypervariable regions of the bacterial 16S gene have been amplified and sequenced. The OTUs identified by the different pipelines were classified to known genera using RDP^6^ (confidence level ≥ 90%), discarding those that could not be classified, thus guaranteeing that low quality OTUs or chimaeras that might have not been identified in the filtering step did not influence the results. Counting the number of distinct genera guarantees that novel OTUs deriving from the artificial splitting of single taxa do not bias our measure of sensitivity. Results are shown in Figure 2 and in Supplementary Table S3. In all cases, we found that MICCA estimates a number of OTUs that is intermediate between what found by QIIME and UPARSE (always much closer to the UPARSE estimate), as already found on the synthetic datasets. However, the number of distinct genera is always only marginally smaller than the estimate given by QIIME, showing that the higher selectivity in the OTUs identification does not affect the ability of MICCA to characterize the biological complexity of the analysed samples. These results (number of OTUs and number of distinct genera) indicate that MICCA reaches a good compromise between the need to guarantee the quality of the OTUs and to identify less abundant taxa, without excessive loss of information due to filtering, thus confirming the advantage of MICCA over competing software.

### Estimates of the diversity indices

**Figure 3:**
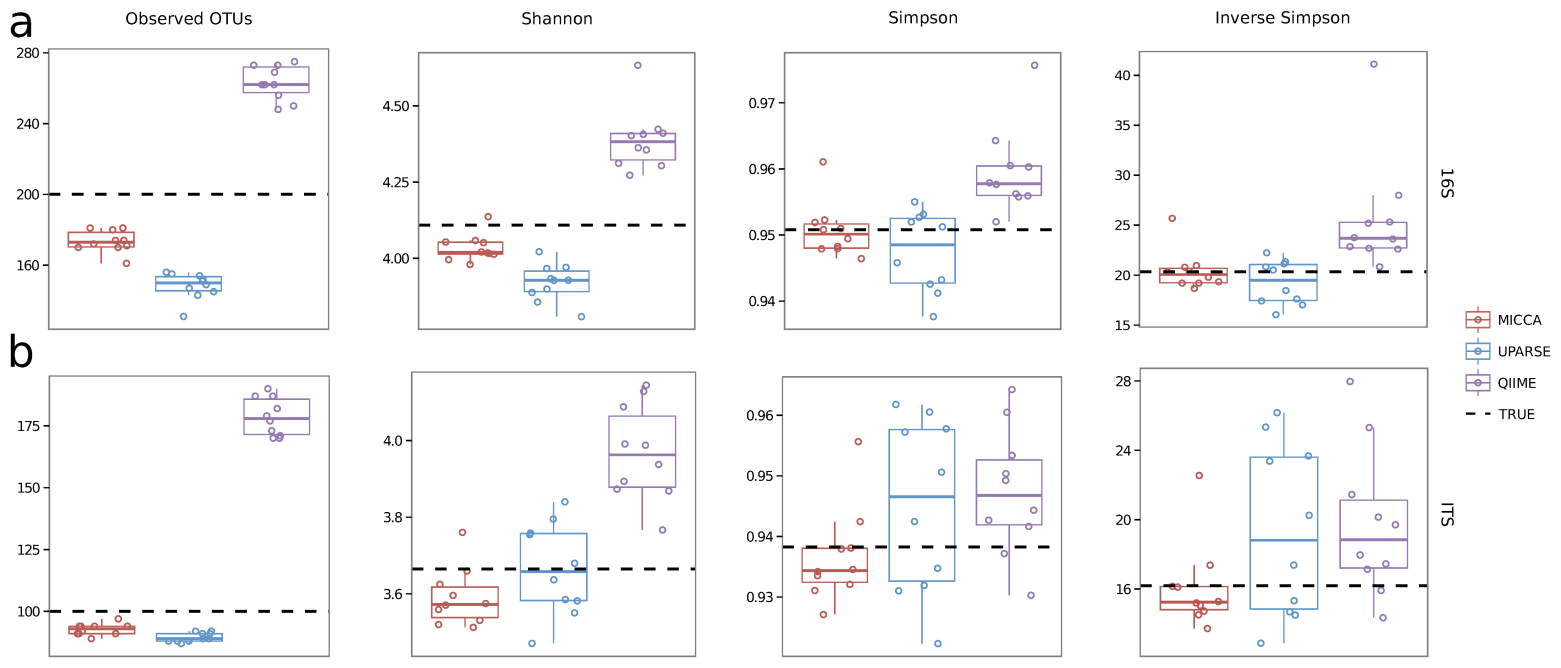
Diversity indices computed using MICCA, UPARSE and QIIME on the 16S-10 (above) and ITS-10 (below) simulated dataset. The dashed lines represent the real values.

To assess the stability of the OTUs estimates and of various ecological measure of complexity, we generated two independent datasets, one for 16S bacterial amplicons (16S-10) and one composed by ITS fungal amplicons (ITS-10). Each dataset was composed by 10 independent samples having the same community structure and number of OTUs. Results are shown in Figure 3a and 3b for the 16S-10 and ITS-10 datasets respectively, where the horizontal lines indicates the median over the replicated datasets and the dashed lines indicates the real values. The impact of these different reconstructions of the microbial communities is immediately evident calculating the Shannon, Simpson and Inverse Simpson diversity indices, commonly used in environmental metagenomic studies to measure the complexity of the microbial communities. With the exception of the Shannon diversity index for the ITS-10 dataset, the median of the indices estimated using MICCA are closer to the real value than the indices estimated with the other two pipelines. Moreover, the spread in terms of interquartile range (IQR) of the estimates obtained by MICCA is always considerably lower than the dispersion of the estimates obtained both with UPARSE and QIIME (Supplementary Table S4).

### Performances on biological datasets

**Figure 4:**
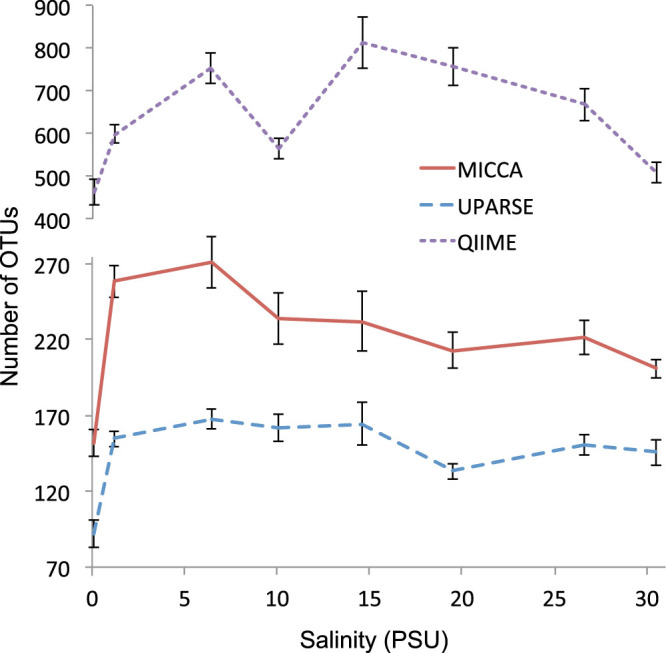
Curves of the salinity dataset after pooling and rarefaction. The plot represents the variation of the number of OTUs as a function of salinity in marine water in the Delaware Bay obtained analysing the data using the three pipelines, MICCA, UPARSE and QIIME.

To show the effect of the MICCA pipeline on real data, we have analysed two public metagenomic datasets, one reporting the bacterial composition of samples collected along the Delaware Bay, and the other reporting the bacterial component of the distal gut microbiota of three individuals in a time series including two courses of antibiotic treatment with ciprofloxacin. The analysis of an environmental dataset and a gut dataset puts MICCA to the test of two of the most common applications of metagenomics. The Delaware Bay study is focused on the analysis of diversity, composition and activity of bacteria in a salinity gradient typical of estuarial environment. We have evaluated the Chao1 estimator of the number of OTUs after sample pooling and rarefaction using MICCA, UPARSE and QIIME. The results are shown in Figure 4. Qualitatively the results confirm the behaviours of the three pipelines observed on the simulated datasets. QIIME finds more than twice as many OTUs as identified by MICCA, while UPARSE is much more conservative and individuates less OTUs in each of the different samples.

**Figure 5:**
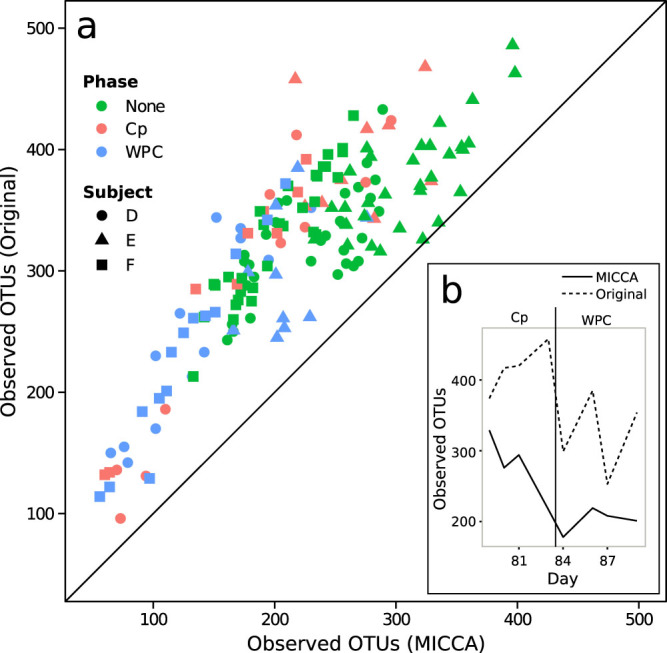
(a) Scatter plot of the number of OTUs identified by the analysis pipeline used in the paper of Dethlefsen et al. for patients, D, E and F vs the number of OTUs estimated by MICCA. (b) Patient E. The microbial growing curve inferred by the two pipelines during the first antibiotic ciprofloxacin (Cp) treatment and the week post Cp (WPC).

Analysis of the antibiotic treatments study highlights the advantages of using MICCA in term of the higher number of sequences used and more consistent estimates of the number of OTUs across replicates. In the original study a custom pipeline based on mothur and UCLUST with a reference-based clustering protocol was applied^7^. In this protocol, sequences that did not have a hit with a reference OTU were excluded from the analysis. After pre-processing, clustering and rarefaction, the number of reads per sample obtained by using the MICCA pipeline was 3.2 × 10^6^ clustered into 1466 OTUs, while the reads per sample used in Ref. 7 was 1.8 × 10^6^ clustered into 2582 OTUs. By aligning the representative sequences of the OTUs obtained in Ref. 7 against the representative sequences of the OTUs inferred by MICCA, we found that 1395 original OTUs aligned to 702 OTUs of MICCA (alignments performed using blastn and considering only hits with identity >0.97 and alignment length >100 bp) (see Figure 5a). The more robust estimate of the number of OTUs improves the consistency of the results at the different time points. For instance, in the case of patient E, first Cp treatment, (Figure 5b) it is worth noting that, as expected, the number of OTUs estimated by MICCA decreases during the first antibiotic treatment (solid line, days 79-83), while we observe an inverse trend with the analysis pipeline used in the paper of Dethlefsen (dashed line), probably due to a less robust estimate of the number of OTUs. A generally lower number of OTUs and smoother time series are obtained also for the other patients (patients D, E and F in Supplementary Figures S5, S6, S7 respectively).

## Discussion

The increasing throughput of NGS technologies and the decreasing costs of sequencing is triggering an explosion of the number of projects that use metagenomics to characterize the structure and composition of microbial communities. However, while standard experimental protocols and technologies are emerging that allow the comparison of raw datasets generated by different groups, the reusability of the processed data is often hampered by the lack of standard processing and analysis pipelines. In addition, existing software are often complex to use and allow many different combinations of parameters that make it difficult to document and compare results from different experiments. The availability of standard, easy-to-use and optimized software pipelines would greatly benefit the scientific community involved in the study of the microbiota using metagenomic technologies.

The analysis of metagenomic sequences can be divided into three main steps: i) quality filtering of the reads and chimera detection; ii) OTU clustering; iii) OTU annotation and analysis. While the third step relies on reference sequence databases to define the taxonomic structure of the sample, the first two steps mostly depend on complex computational procedures for quality filtering and clustering and are critical in order to produce consistent data. For example, failing to eliminate spurious reads might lead to an overestimate of the sample complexity, while a stringent filtering process might eliminate too many reads penalizing the least abundant species. One example of this behaviour is represented by UPARSE, which implements a stringent quality filtering algorithm at the cost of discarding a large fraction of the sequence data. MICCA, implementing a more flexible filtering strategy, tends to use more efficiently the sequenced reads and therefore provides a more realistic estimate of the sample complexity.

The second step of the procedure, the clustering process, is also critical. The use of greedy clustering algorithms, while being capable to quickly analyse large datasets, might lead to a definition of OTUs that overestimates the number of different species actually present in the sample. One example of this is represented by UCLUST algorithm exploited by QIIME, which systematically overestimates the sample richness, failing to converge to a finite number of OTUs in rarefaction analysis. This feature, share by mothur, makes it difficult to merge samples sequenced to different depths and might lead to sequencing to excessive depth thus increasing the experimental costs. A more accurate approach for the choice of the centroid is implemented in the UPARSE and MICCA pipelines, starting the clustering process from the most abundant reads individuated after the dereplication step. In this way UPARSE and MICCA, using abundance information in the clustering step, are able to provide estimates of the number of OTUs that are less dependent on sequencing depth, thus allowing to attain a good description of metagenomic samples with lower sequencing depth than needed by QIIIME and mothur. In addition, the efficient use of sequence data identifies a richer population structure than UPARSE, avoiding the danger of filtering out the complexity of the sample.

Given the lack of official and well-studied datasets to test the analysis tools, it is generally difficult to assess the accuracy and reliability of a metagenomic pipeline. To overcome these limitations we tested our pipeline on simulated data where the OTU composition was known, showing that MICCA improves over the existing software. MICCA was able to accurately reconstruct the OTU composition of the samples and gave reliable estimate of the most commonly used diversity indices. In addition, the estimated OTUs, although less numerous that what obtained by QIIME, covered a similar range of biological diversity, as witnessed by the similar number of identified bacterial genera in samples derived from the HMP. When tested on real datasets representing the two most common scenarios for metagenomics studies, *i.e.* environmental metagenomics and gut metagenomics, MICCA showed a higher consistency of the results. For instance, MICCA was able to reconstruct the expected growing curve of the microbiota during two antibiotic courses even in the absence of a reference database for OTUs. This suggests that MICCA can be effectively used also on samples that are rich in species not previously characterized, as is often the case in environmental metagenomic projects.

## Methods

### MICCA

MICCA is an open-source software pipeline built using the Python and C programming languages (https://www.python.org/) and several external applications. Starting from raw sequencing data, MICCA performs a series of initial analyses to clean the reads cutting the primers and trimming the low quality regions. The pre-processing module of MICCA implements a sliding window approach for quality filtering, discarding the 3′-end of the read when the average quality in the sliding window (including 10% of the read length) drops below a specified threshold. Moreover, ending and contiguous Ns are discarded. After trimming, sequences are discarded if their length is below a specified threshold. All the preprocessing steps are performed by a single command line tool (micca-preproc) which wraps cutadapt^8^ for primers trimming (modified to include IUPAC codes support) and sickle (https://github.com/najoshi/sickle) for the windowed adaptive quality filtering. After that, a de novo clustering (micca-otu-denovo, using OTUCLUST, a clustering algorithm specifically designed for MICCA) or a reference-based clustering (micca-otu-ref, which wraps DNACLUST^9^) can be performed. For the chimera-filtering step, OTUCLUST embeds the public domain version of UCHIME^10^. In de novo mode, the representative sequences of the OTUs can finally be classified using BLAST+ (against a QIIME-formatted database, such as Greengenes^11^, UNITE^12^) or exploiting the RDP classifier^6^ (version 2.6+ are supported). Furthermore, the single command micca-phylogeny produces a multiple alignments using MUSCLE^13^, T-Coffee^14^ or PyNAST^15^ and infers a phylogenetic tree with FastTree^16^. In Supplementary Table S5 we reported the computation times of the MICCA pipeline using different wrapped tools available in each processing step.

MICCA can be easily used on a wide range of *NIX platforms, from laptops to high performance computing clusters. The output of MICCA is fully compatible with the R package phyloseq^17^ for downstream analyses.

### OTUCLUST

OTUCLUST is a new open-source application specifically created to partition a set of amplicon reads into clusters of sequences within a given identity threshold. OTUCLUST is the default clustering method used in micca-otu-denovo. In OTUCLUST, a cluster is built starting from a centroid (the representative sequence) and from the sequences which have pair-wise similarity with the representative one above the threshold.

### Overview of OTUCLUST algorithm

The OTUCLUST algorithm can be divided into three main steps: a) dereplication and abundance estimation, b) de novo chimera removal (optional) and c) clustering using a greedy approach, where representative sequences are selected starting from high-abundance reads, which are more likely to be representative sequences (Supplementary Figure S1).

The aim of the dereplication is the removal of duplicate sequences. This step is performed by the clustering procedure (described below) with an identity threshold of 100%. Given the increasing size of metagenomic datasets, this step is computationally intensive. In order to provide a faster alternative we have included the possibility to use an exact prefix matching algorithm (MICCA-FAST). Abundances of the dereplicated sequences are estimated by counting the number of reads. Dereplicated sequences are ordered by their abundance and passed to UCHIME^10^ for chimera detection. Reads detected as chimeras are removed.

Dereplicated and chimera-free sequences, ordered according to their abundance, are used as cluster seeds by the clustering algorithm. The clustering procedure relies on a search algorithm defined as follows:

Inputs:

- Query sequence: a cluster seed (dereplicated sequence)
- Sequence database: all sequences

- Given a query sequence Q, the sequence database is sorted by decreasing k-mer similarity. The k-mer similarity is defined as in Ref. 13:
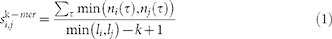
where *τ* is a k-mer, *n* is the number of occurences of the k-mer in the sequences and *l_i_*, *l_j_* are the lenght of sequences *i* and *j* respectively.
- For each sequence in the sorted database (target sequence, T) the similarity 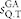 is computed using the Needleman–Wunsch algorithm. If 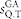 is greater or equal than the identity threshold *s*^thr^ (e.g. 0.97) the sequence Q is added to the results and the reject counter *n*_rej_ is set to zero, otherwise *n*_rej_ is incremented by one.*Definition of pair-wise similarity* 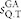. Given a global alignment (GA), between the sequences *i* and *j*, the pair-wise identity is defined as:
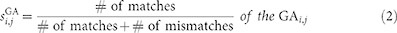
Internal and external gaps are ignored in the definition of 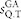
18, and mismatch and gap penalties are set equal to one in the GA.
- If *n*_rej_ is above the given threshold *m* (a value of 32 has proven to be a good choice for this threshold) it is unlikely that another hit exists. In this case the reads belonging to the cluster are removed from the sequence database and a new seed sequence is chosen according to its abundance.

### Synthetic data

We generated two amplicon datasets for the V3-V5 variable region of the16S rRNA gene for bacteria (datasets 16S-R and 16S-10) and one dataset for the ITS (ITS2) region for fungi, mimicking data obtained from a Life Sciences 454 sequencer (GS FLX Titanium series reagents) using Grinder^19^ starting from 99% clustered OTUs in Greengenes^11^ version 13_05 for the 16S data and 99% clustered OTUs in the UNITE database^12^ release 04_07_2014 (after removing duplicate sequences) for the ITS datasets (details, commands and Grinder profile files in Supplementary Data). Quality profiles of the reads of the 454 pyrosequencing process were simulated with flowsim^20^. Community structures were simulated with a power law rank-abundance distribution and 20% of chimeras were generated (90% bimeras, 10% trimeras).

#### 16S-R

30 samples were generated from a single Grinder run (100000 reads, 500 OTUs). After the application of the flowsim model, samples were rarefied to a fixed depth at steps of 1000 reads, obtaining 30 samples containing between 1000 and 30000 sequences (dataset available at http://compmetagen.github.io/micca/data/16S-R.tar.bz2).

#### 16S-10

10 independent samples containing 5000 sequences were generated using Grinder (200 OTUs) and flowsim (dataset available at http://compmetagen.github.io/micca/data/16S-10.tar.bz2).

#### ITS-10

10 independent samples containing 5000 sequences were generated using Grinder (100 OTUs) and flowsim (dataset available at http://compmetagen.github.io/micca/data/ITS-10.tar.bz2).

### Biological data

#### HMP dataset

We analysed 12 samples of the Human Microbiome Project^5^ dataset where the V1-V3, V3-V5 and V6-V9 hypervariable regions of the bacterial 16S gene have been amplified and sequenced using the Roche-454 FLX Titanium platform. Details and NCBI accession numbers are available in the Supplementary Table S6.

#### Salinity dataset

This dataset characterizes the bacterial communities along the salinity gradient in the Delaware Bay and it is described in Ref. 21. The 454 GS FLX Titanium “whole water” samples were downloaded from the NCBI Sequence Read Archive database, accession number SRA052537 (samples FB_WD, Bay_WD, X14_WD, X16_WD, X18_WD, X20_WD, X22_WD, X26_WD, X28_WD).

#### Antibiotic dataset

Dataset of distal gut of three adult healthy subjects obtained by collecting stool samples (52–56 per subject) over a 10-mo interval, during which time these subjects took two 5-d courses of the antibiotic ciprofloxacin (Cp) separated by 6 mo. 454 GS FLX Titanium data from NCBI Sequence Read Archive, accession number SRA020961^7^.

### Software availability

MICCA is an Open Source project and it is freely available at http://compmetagen.github.io/micca/.

## Additional Information

**How to cite this article**: Albanese, D., Fontana, P., De Filippo, C., Cavalieri, D. & Donati, C. Serum FGF21 levels are associated with brown adipose tissue activity in humans. *Sci. Rep.* **5**, 9743; doi: 10.1038/srep09743 (2015).

## Supplementary Material

Supplementary Information Supplementary Info

Supplementary Information Supplementary Table S3

Supplementary Information Supplementary Table S6

## Figures and Tables

**Table 1:** List of the software wrapped in the main tools available in the MICCA pipeline

Command	Description	Tools	Notes
micca-preproc			supports gapped alignment and IUPAC codes for primer trimming
	• primer trimming both in the 5′ and 3′ ends of reads using semi-global alignments	• Cutadapt	
	• quality trimming using sliding windows	• SICKLE	
	• minimum length filtering		
micca-otu-denovo			BLAST+: Greengenes, Silva and UNITE QIIME-formatted databases are supported. RDP: versions 2.6+ are supported.
	• de novo sequence clustering	• OTUCLUST	
	• de novo chimera filtering	• UCHIME	
	• taxonomic assignment with RDP classifier or BLAST+	• RDP Classifier	
		• BLAST+	
micca-otu-ref			Greengenes, Silva and UNITE QIIME-formatted databases are supported
	• reference-based clustering	• DNACLUST	
micca-phylogeny			
	• de novo and template-based multiple sequence alignment (MSA)	• MUSCLE	
	• phylogenetic tree reconstruction	• T-Coffee	
		• PyNAST	
		• FastTree	

**Table 2:** Comparison of MICCA, QIIME and UPARSE on both 16-10 and ITS-10 datasets in terms of average % of reads passing the quality filtering step, number of OTUs found, chimeric and redundant OTUs (i.e. OTUs corresponding to the same centroid in the synthetic datasets). Standard deviations are indicated in parentheses

Dataset	Pipeline	% reads passing the filtering step	OTUs found	% Chimeric Reads	Chimeric OTUs	Redundant OTUs
16S-10	MICCA	86.6 (1.8)	173.4 (6.2)	13.1 (0.7)	0.5 (0.5)	0 (0)
	QIIME	84.4 (2.1)	263.3 (9.6)	12.0 (0.6)	26.8 (5.8)	31.5 (3.4)
	UPARSE	64.6 (2.4)	148.3 (7.4)	15.5 (0.8)	0.2 (0.4)	0 (0)
ITS-10	MICCA	74.1 (5.2)	93 (2.3)	12.7 (0.5)	0.6 (0.8)	0 (0)
	QIIME	71.8 (5.5)	179.1 (7.5)	11.5 (0.5)	28.7 (10.4)	34 (2.3)
	UPARSE	66.8 (4.1)	89.5 (1.8)	15.5 (0.6)	0.1 (0.3)	0.9 (0.7)
