# Supplementary material for: MICCA: a complete and accurate software for taxonomic profiling of metagenomic data
Source: Sci Rep. 2015 May 19;5:9743. doi: 10.1038/srep09743 (PMC4649890; doi:10.1038/srep09743)
Supplement: Supplementary Information — Supplementary Info [file srep09743-s1.pdf]

Supplementary Material for

MICCA: a complete and accurate software for taxonomic profiling of metagenomic data

Davide Albanese[1,§], Paolo Fontana[1,§], Carlotta De Filippo[2], Duccio Cavalieri[1,*] and Claudio Donati[1,*]

[1]Fondazione Edmund Mach, Research and Innovation Centre, Computational Biology Department, Via E. Mach 1, 38010 - S. Michele all'Adige (TN), Italy.

[2]Fondazione Edmund Mach, Research and Innovation Centre, Food Quality Nutrition & Health Department, Via E. Mach 1, 38010 - S. Michele all'Adige (TN), Italy.

* Correspondence to: Tel: +39 0461 615 696; Email: claudio.donati@fmach.it. Correspondence may also be addressed to duccio.cavalieri@fmach.it.

[§] The authors wish it to be known that, in their opinion, the first two authors should be regarded as joint First Authors.

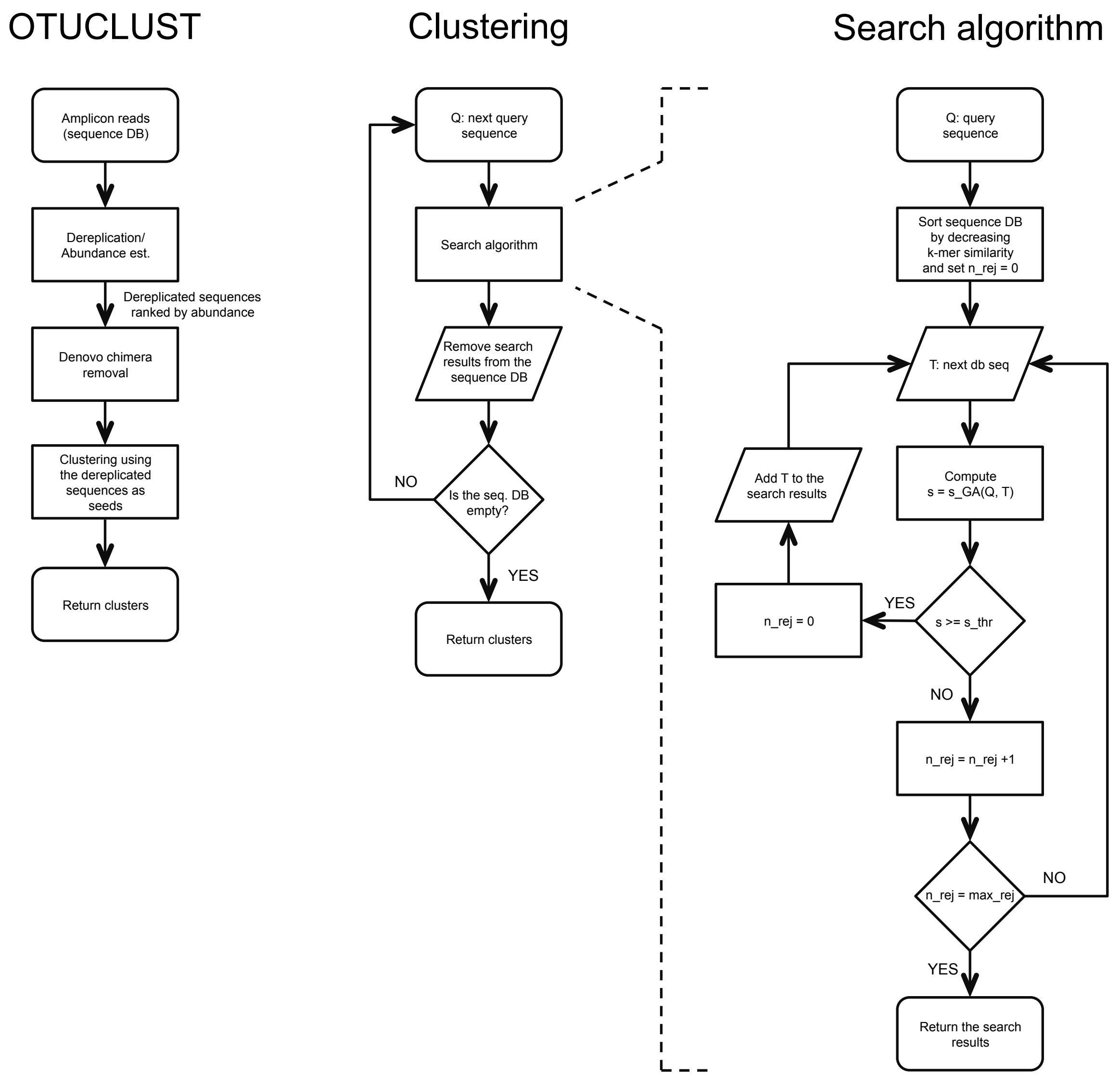

Figure S1: Overview of OTUCLUST algorithm. Left: the algorithm can be divided into three main steps: a) dereplication and abundance estimation, b) de novo chimera removal (optional) and c) clustering using a greedy approach, where representative sequences are selected starting from high-abundance reads (which are more likely to be true seed sequences). Middle: Schema of the clustering procedure. Right: the search algorithm implemented in OTUCLUST, where global alignments are computed according to the k-mer similarity ranking.

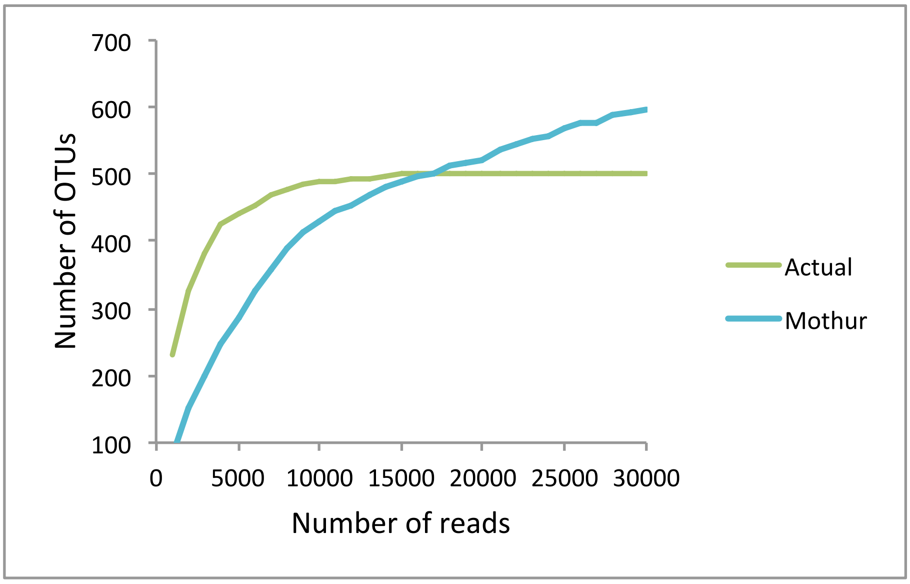

Figure S2: Estimated number of OTUs obtained by Mothur on the 16S-R synthetic dataset as a function of the rarefaction depth. Similarly to QIIME, Mothur does not converge to a finite value in the simulated range.

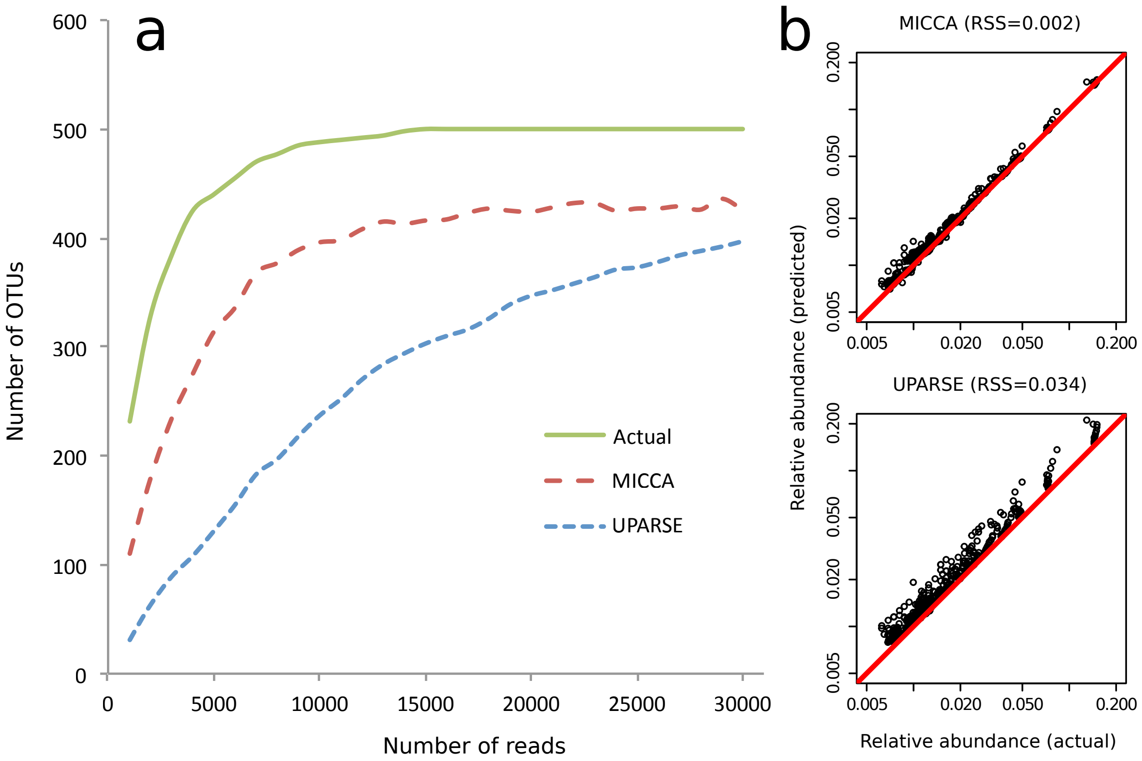

Figure S3: Evaluation of MICCA pipeline performance compared with UPARSE on the 16S-R dataset with reads truncated at 350 bp. Analyses were performed skipping the quality filtering steps. The continuous green lines represent the real values. In (a) the rarefaction curves are plotted. In (b) the relative abundances of the top 20 ranked OTUs compared to the actual values. RSS: Residual Sum of Squares.

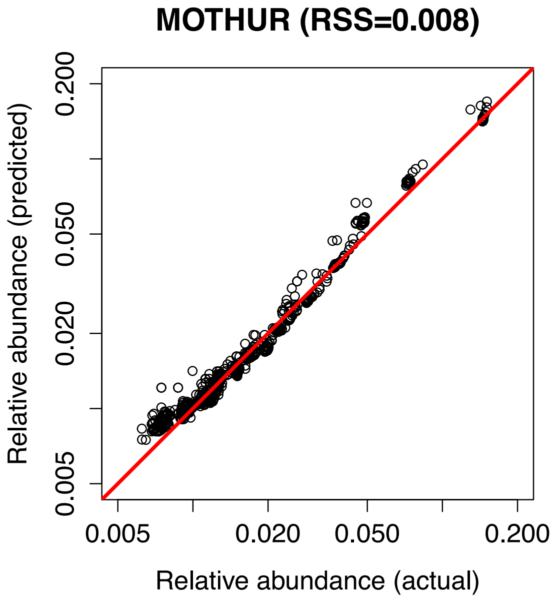

Figure S4: Relative abundances of the top 20 most abundant OTUs in the synthetic dataset estimated by mothur, plotted against the effective abundances. The Residual Sum of Squares (RSS) for mothur is 0.008, t twice the value obtained by MICCA (0.004), but smaller than the values produced by QIIME and UPARSE (0.027 and 0.028, respectively).

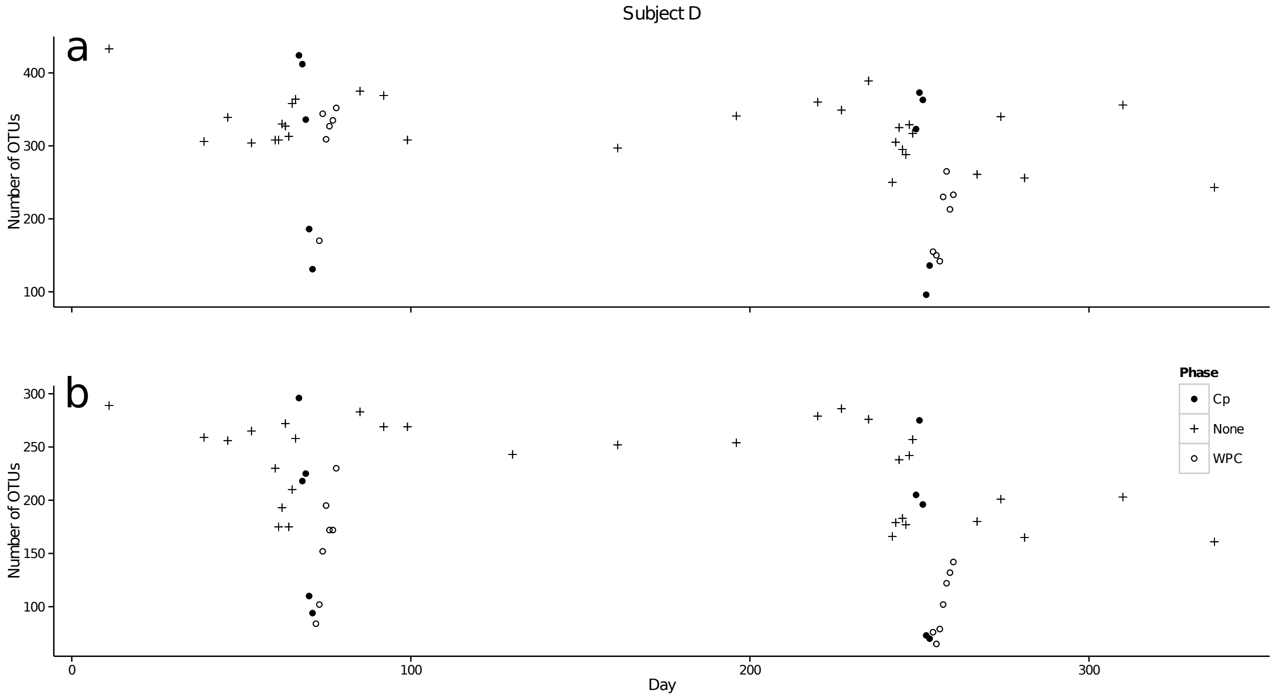

Figure S5: Subject D. Number of OTUs estimated by (a) MICCA (b) Dethlefsen et al. (1). Filled points represent the 5-d of the antibiotic ciprofloxacin (Cp) courses, empty circles represent the weeks post Cp (WPC).

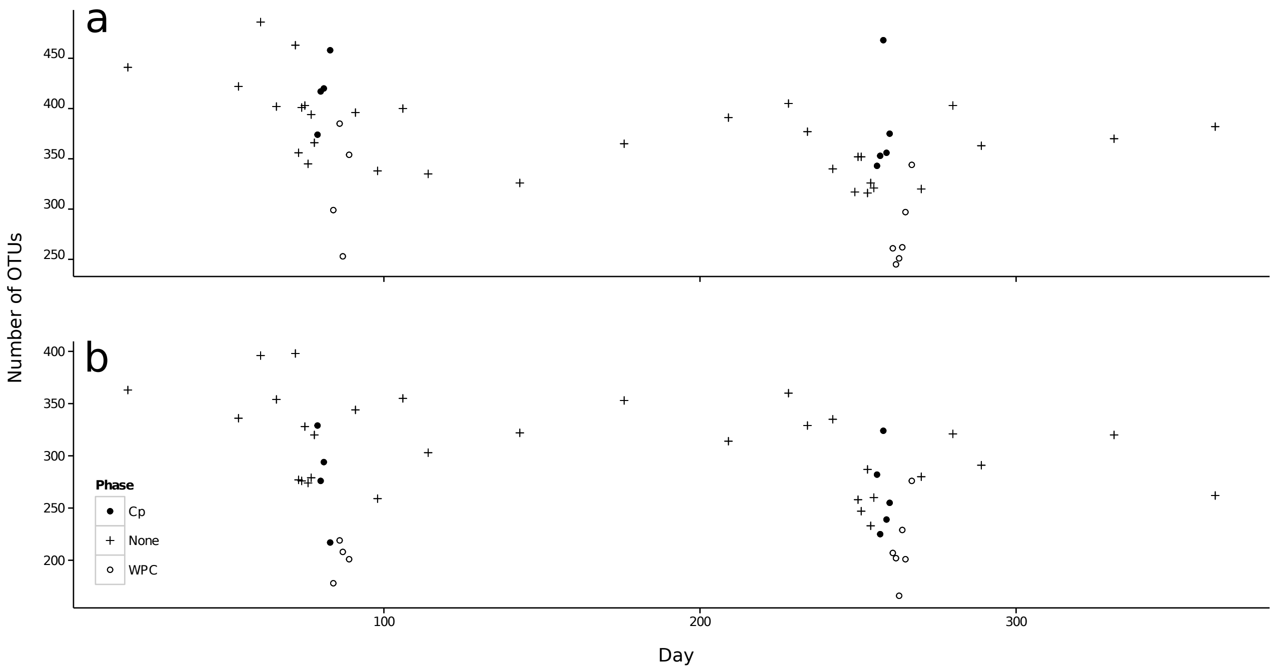

Figure S6: Subject E. Number of OTUs estimated by (a) MICCA (b) Dethlefsen et al. (1). Filled points represent the 5-d of the antibiotic ciprofloxacin (Cp) courses, empty circles represent the weeks post Cp (WPC).

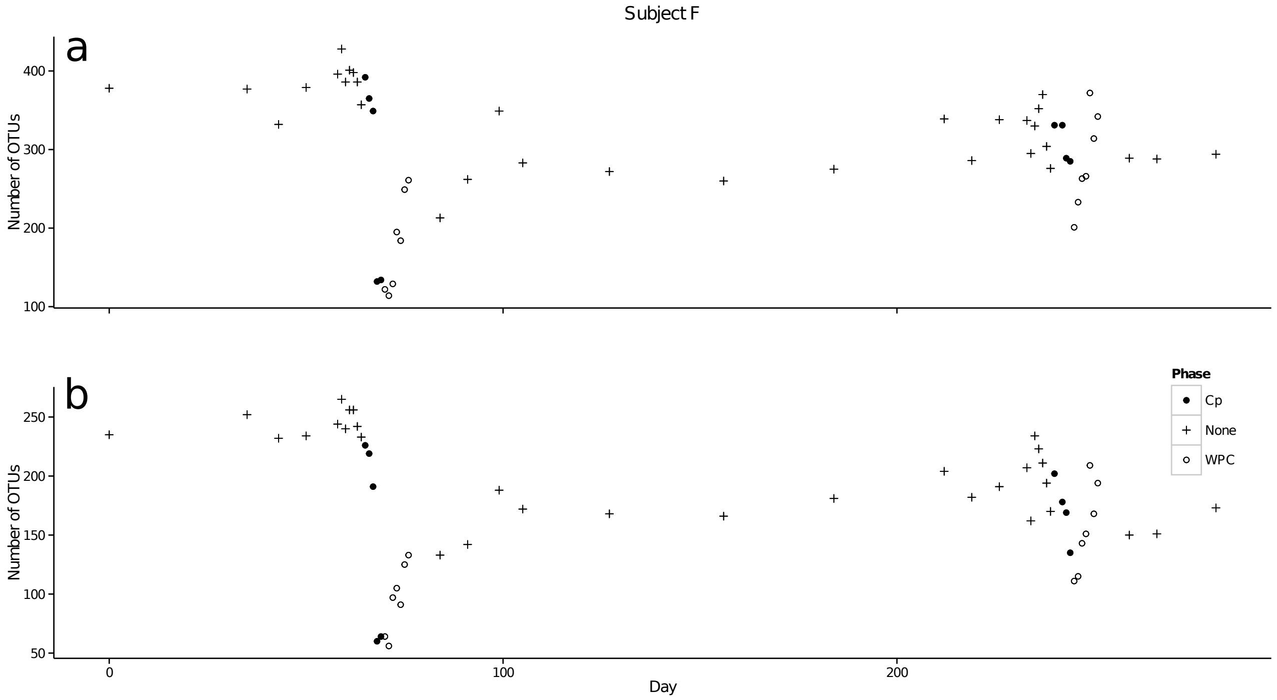

Figure S7: Subject F. Number of OTUs estimated by (a) MICCA (b) Dethlefsen et al. (1). Filled points represent the 5-d of the antibiotic ciprofloxacin (Cp) courses, empty circles represent the weeks post Cp (WPC).

N READS	Actual	MICCA	MICCA FAST	UPARSE	QIIME	MOTHUR
1000	231	94	58	54	114	81
2000	328	167	116	106	212	150
3000	384	218	152	146	277	201
4000	425	264	195	182	334	247
5000	440	298	228	217	395	286
6000	455	328	252	238	440	328
7000	470	345	288	260	489	360
8000	477	360	294	268	514	392
9000	485	380	313	276	549	414
10000	488	370	321	292	572	430
11000	490	393	340	310	602	444
12000	492	394	347	324	624	455
13000	494	396	360	335	645	468
14000	498	404	367	341	672	481
15000	500	411	369	340	701	490
16000	500	405	383	349	713	497
17000	500	419	376	357	730	503
18000	500	405	385	367	742	514
19000	500	418	387	371	763	519
20000	500	423	387	374	781	522
21000	500	420	397	377	794	538
22000	500	423	397	376	826	545
23000	500	418	394	378	846	552
24000	500	418	388	376	858	556
25000	500	423	400	381	892	569
26000	500	430	406	385	906	575
27000	500	419	405	387	925	577
28000	500	422	406	390	946	587
29000	500	429	417	388	970	591
30000	500	419	403	390	992	598

Table S1: 16S-R dataset. Number of OTUs estimated by MICCA and MICCA-FAST, with the latter implementing an exact string matching dereplication algorithm (see OTUCLUST algorithm), as a function of the size of the rarefied sample.

N READS	Actual	MICCA	MICCA FAST	UPARSE	QIIME	MOTHUR
1000	4.417	3.724	3.284	3.334	3.986	3.667
2000	4.575	4.125	3.839	3.777	4.407	4.060
3000	4.597	4.243	3.956	3.941	4.519	4.180
4000	4.629	4.334	4.097	4.064	4.598	4.312
5000	4.635	4.395	4.193	4.179	4.716	4.388
6000	4.659	4.446	4.267	4.253	4.812	4.475
7000	4.676	4.484	4.353	4.291	4.852	4.528
8000	4.686	4.497	4.374	4.309	4.882	4.573

9000	4.694	4.536	4.410	4.327	4.921	4.607
10000	4.702	4.509	4.444	4.364	4.950	4.625
11000	4.708	4.553	4.484	4.408	4.962	4.631
12000	4.710	4.545	4.462	4.421	4.967	4.642
13000	4.715	4.533	4.583	4.439	5.001	4.651
14000	4.719	4.561	4.523	4.450	5.016	4.660
15000	4.722	4.569	4.528	4.445	5.010	4.681
16000	4.723	4.565	4.632	4.460	5.014	4.684
17000	4.727	4.597	4.531	4.480	5.018	4.687
18000	4.728	4.562	4.506	4.513	5.012	4.699
19000	4.735	4.585	4.531	4.527	5.030	4.701
20000	4.741	4.589	4.590	4.536	5.038	4.708
21000	4.744	4.604	4.558	4.546	5.041	4.724
22000	4.745	4.595	4.588	4.543	5.068	4.726
23000	4.741	4.589	4.547	4.543	5.071	4.724
24000	4.738	4.586	4.536	4.536	5.075	4.720
25000	4.738	4.593	4.538	4.544	5.122	4.724
26000	4.737	4.605	4.646	4.547	5.106	4.726
27000	4.736	4.588	4.560	4.542	5.110	4.726
28000	4.740	4.590	4.558	4.548	5.118	4.736
29000	4.745	4.606	4.582	4.550	5.126	4.738
30000	4.745	4.596	4.573	4.551	5.132	4.737

Table S2: *16S-R* dataset. Shannon diversity indexes estimated by MICCA and MICCA-FAST, with the latter implementing an exact string matching dereplication algorithm (see OTUCLUST algorithm), as a function of the size of the rarefied sample.

Table S3 (external file): Diversity indices, number of genera and families (>=90% RDP classifier confidence) detected using MICCA, UPARSE and QIIME on the HMP dataset.

Dataset	Pipeline	Observed		Shannon		Simpson		Inverse Simpson	
		Median	IQR	Median	IQR	Median	IQR	Median	IQR
16S-10	TRUE	200	-	4.109	-	0.951	-	20.327	-
	MICCA	**173**	**8.250**	**4.019**	**0.039**	**0.950**	**0.004**	**20.056**	**1.454**
	UPARSE	150	8.000	3.928	0.068	0.948	0.010	19.480	3.595
	QIIME	262	14.500	4.383	0.087	0.958	**0.004**	23.685	2.558
ITS-10	TRUE	100	-	3.665	-	0.938	-	16.197	-
	MICCA	**93**	**3.000**	3.573	**0.080**	**0.934**	**0.006**	**15.237**	**1.339**
	UPARSE	89	**3.000**	**3.658**	0.175	0.947	0.025	18.814	8.756
	QIIME	178	14.250	3.963	0.185	0.947	0.011	18.834	3.909

Table S4: Diversity indices computed using MICCA, UPARSE and QIIME on both the 16S-10 and ITS-10 simulated dataset. The numbers in bold represent either the closest values to the true one (median) or the smaller inter-quantile range (IQR).

Action	Command	Real [s]	User [s]	Sys [s]
Preprocessing	`micca-preproc -f TACGGYTACCTTGTTAYGACTT -O 15 -q 20 -l 300 attached_keratinized_gingiva.fastq -o pre`	9.67	9.55	0.09
OTU clustering and taxonomy assignment with the RDP classifier	`micca-otu-denovo pre/attached_keratinized_gingiva.fastq -t rdp —s 0.97 —c —o otus_denovo_rdp`	120.06	120.61	1.59
OTU clustering and taxonomy assignment with BLAST+	`micca-otu-denovo pre/attached_keratinized_gingiva.fastq -t blast -s 0.97 -c -o otus_denovo_blast --blast-ref greengenes_2013_05/rep_set/97_otus.fasta --blast-ref-taxonomy greengenes_2013_05/taxonomy/97_otu_taxonomy.txt --blast-num-threads 4`	208.40	402.14	1.74
Multiple alignment with	`micca-phylogeny otus_denovo_rdp/representatives.fasta -a denovo_muscle -o phylo_muscle`	1.95	1.89	0.03

MUSCLE and tree reconstruction with FastTree				
Multiple alignment with T-Coffee and tree reconstruction with FastTree	`micca-phylogeny otus_denovo_rdp/representatives.fasta -a denovo_tcoffe --tcoffe-num-threads 4 -o phylo_tcoffe`	41.00	138.63	0.54
Multiple alignment with PyNAST and tree reconstruction with FastTree	`micca-phylogeny otus_denovo_rdp/representatives.fasta -a template -o phylo_pynast --template-min-perc 75 --template-file greengenes_2013_05/rep_set_aligned/97_otus.fasta`	128.39	123.18	3.65

Table S5: Performances of MICCA pipeline with different combinations of the processing steps. Real, User and System time are reported. The computer used is a workstation based on Intel® Core™ i7-3770 CPU @ 3.40 GHz x 8, 16 GB RAM, with Ubuntu 13.04, Cutadapt 1.4.2, Sickle 1.210, RDP 2.8, BLAST 2.2.28+, MUSCLE 3.8.31, T-Coffe 20140618_11:18, PyNAST 0.1, FastTree 2.1.3.

Table S6 (external file): Metadata and NCBI accession numbers for the HMP dataset.

16S-R SIMULATED DATA GENERATION

```
# 16S-R Grinder profile file
-reference_file 99_otus.fasta
-total_reads 100000
-read_dist 450 normal 50
-forward_reverse primers_16S.fasta
-unidirectional 1
-length_bias 0
-copy_bias 0
-chimera_perc 20
-chimera_dist 90 10
-chimera_kmer 0
-abundance_model powerlaw 1
-num_libraries 1
-multiplex_ids barcodes.fasta
-diversity 500
-random_seed 1
-qual_levels 30 10
-fastq_output 0
-base_name 16S
-output_dir 16S-R
```

FlowSim command

```
flowsim -G Titanium seqs.fa -o seqs.sff
```

Rarefaction with micca-rarefy-seqs

```
micca-rarefy-seqs seqs.fastq -d NUMBER_OF_SEQS seqs-NUMBER_OF_SEQS.fastq
```

16S-10 SIMULATED DATA GENERATION

```
# 16S-10 Grinder profile file
-reference_file 99_otus.fasta
-total_reads 5000
-read_dist 450 normal 50
-forward_reverse primers_16S.fasta
-unidirectional 1
-length_bias 0
-copy_bias 0
-chimera_perc 20
-chimera_dist 90 10
-chimera_kmer 0
-abundance_model powerlaw 1
-num_libraries 10
-multiplex_ids barcodes.fasta
-diversity 200
-random_seed 1
-qual_levels 30 10
-fastq_output 0
-base_name 16S
-output_dir 16S-10
```

Flowsim command

```
flowsim -G Titanium seqs.fa -o seqs.sff
```

ITS-10 SIMULATED DATA GENERATION

```
# ITS-10 Grinder profile file
-reference_file 99_otus_its.fasta
-total_reads 5000
-read_dist 350 normal 50
-forward_reverse primers_ITS2.fasta
-unidirectional 1
-length_bias 0
-copy_bias 0
-homopolymer_dist balzer
-chimera_perc 20
-chimera_dist 90 10
-chimera_kmer 0
-abundance_model powerlaw 1
-num_libraries 10
-multiplex_ids barcodes.fasta
-diversity 100
-random_seed 1
-qual_levels 30 10
-fastq_output 0
-base_name its
-output_dir its-10
```

Flowsim command

```
flowsim -G Titanium seqs.fa -o seqs.sff
```

PRIMERS FOR 16S-R AND 16S-10 DATASETS

V3-V5 region (Segata et al.. Metagenomic biomarker discovery and explanation)

```
>FWD 357F
CCTACGGGAGGCAGCAG
>REV 926R
CCGTCAATTCMTTTRAGT
```

PRIMERS FOR ITS-10 DATASET

ITS2 region (Shiang Ning Leaw et al.. Identification of Medically Important Yeast Species by Sequence Analysis of the Internal Transcribed Spacer Regions)

```
>FWD ITS3
GCATCGATGAAGAACGCAGC
```

MICCA COMMANDS AND PARAMETERS FOR 16S-R AND 16S-10 DATASETS

Parameter tuning for the preprocessing step is performed trying different combinations of length and quality thresholds on one or more samples, using `micca-preproc-check`. The parameters are chosen balancing the sequence quality and the sensitivity to low abundance species. In this case we choose a quality threshold of 24 and a length threshold of 300 allowing more then 95% of reads passing the filter:

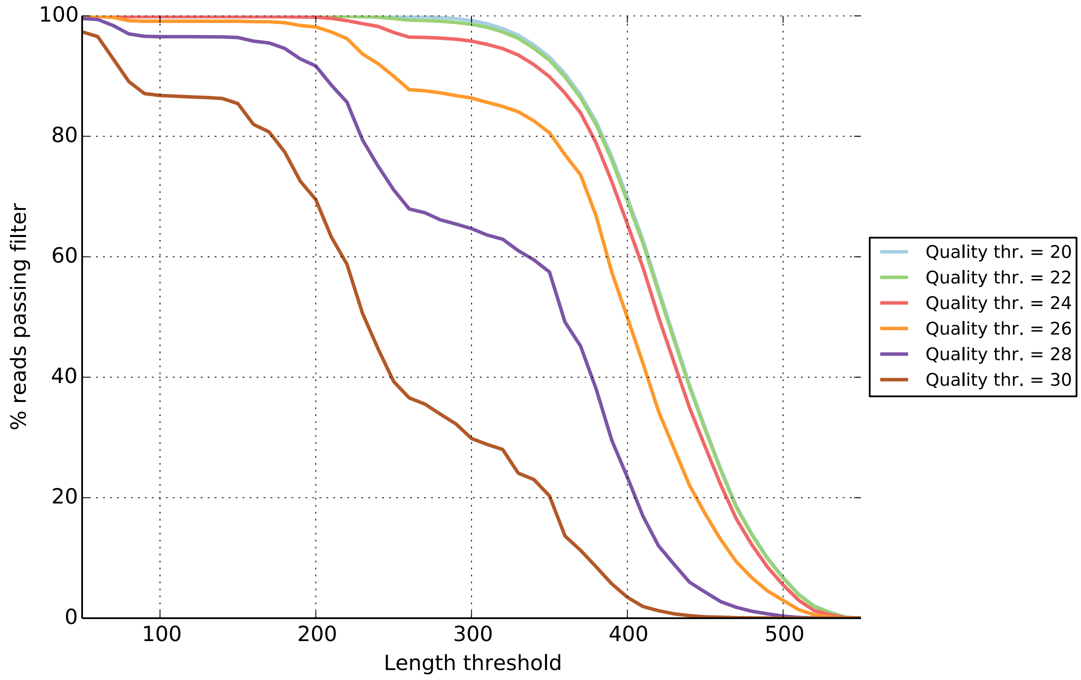

Command lines:

```
# Preprocessing
$ micca-preproc seqs.fastq -f CCTACGGGAGGCAGCAG -r CCGTCAATTCMTTTRAGT -O 15 \
    -q 24 -l 300 -o preproc
# OTU clustering and taxonomy assignment
$ micca-otu-denovo preproc/seqs.fastq -s 0.97 -c -o otus
```

MICCA COMMANDS AND PARAMETERS FOR ITS-10 DATASET

The parameters are chosen balancing the sequence quality and the sensitivity to low abundance species. In this case we choose a quality threshold of 20 and a length threshold of 250 allowing more then 95% of reads passing the filter:

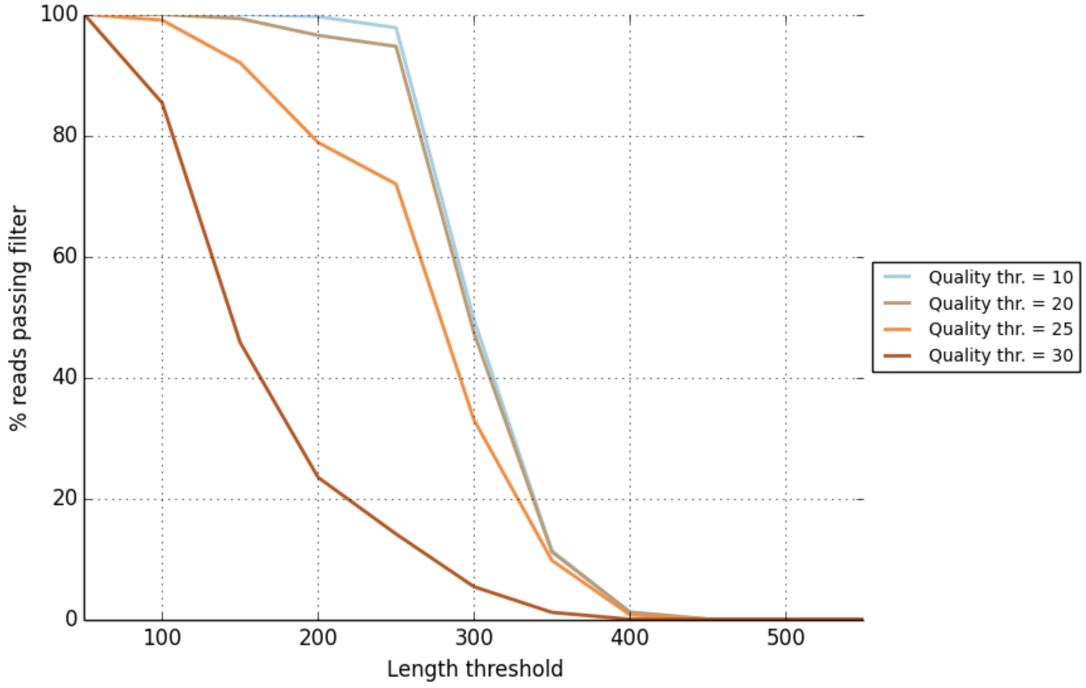

Command lines:

```
# Preprocessing
$ micca-preproc seqs.fastq -f GCATCGATGAAGAACGCAGC -O 15 -q 20 -l 250 -o preproc
# OTU clustering
$ micca-otu-denovo preproc/seqs.fastq -s 0.97 -c -o otus
```

MICCA COMMANDS AND PARAMETERS FOR THE HMP DATASET

```
# Preprocessing
# V1-V3: ATTACCGCGGCTGCTGG
# V3-V5: CCGTCAATTCMTTTRAGT
# V6-V9: TACGGYTACCTTGTTAYGACTT
$ micca-preproc -f ATTACCGCGGCTGCTGG -O 15 -q 20 -l 300 seqs.fastq \
—o preproc
# OTU clustering
$ micca-otu-denovo preproc/seqs.fastq -s 0.97 -c -o otus
```

UPARSE COMMANDS AND PARAMETERS FOR 16S-R AND 16S-10 DATASETS

The UPARSE pipeline is built using the information taken from the Supplementary Material of (2), and commands available at http://drive5.com/usearch/manual/uparse_cmds.html. Parameter tuning on the 16S-10_1 dataset is performed as suggested in (2): the parameters are chosen balancing the sequence quality and the sensitivity to low abundance species. In this case we choose a quality threshold of 3 and a length threshold of 200 allowing more then 70% of reads passing the filter:

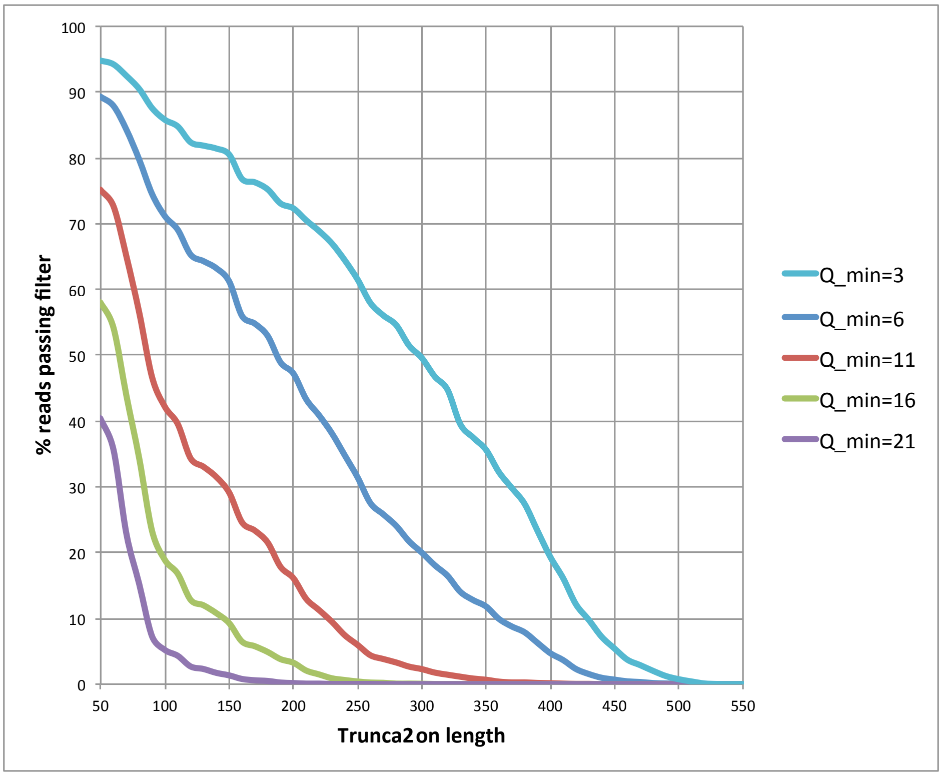

Command lines:

```
$ usearch=./usearch7.0.1090_i86osx32
# Strip barcodes and primers
$ python ./python_scripts/fastq_strip_barcode_relabel2.py seqs.fastq \
    CCTACGGGAGGCAGCAG barcodes.fasta strip > seqs_strip.fastq
# Quality filtering and length truncation
$ $usearch -fastq_filter seqs_strip.fastq -fastaout seqs_filtered.fasta \
    -fastq_truncqual 3 -fastq_trunclen 200
# Dereplication
$ $usearch -derep_fulllength seqs_filtered.fasta -output seqs_derep.fasta \
    -sizeout
# Discard singletons
$ $usearch -sortbysize seqs_derep.fasta -output seqs_sorted.fasta -minsize 2
# UPARSE-OTU
$ $usearch -cluster_otus seqs_sorted.fasta -otus seqs_otus_tmp.fasta
# Label OTU sequences OTU_1, OTU_2...
$ python ./python_scripts/fasta_number.py seqs_otus_tmp.fasta OTU_ > \
seqs_otus.fasta
# Map reads back to OTUs
$ $usearch -usearch_global seqs_filtered.fasta -db seqs_otus.fasta -strand \
plus -id 0.97 -uc seqs_map.uc
```

```
# Create OTU table
$ python ./python_scripts/uc2otutab.py seqs_map.uc > seqs_otu_table.txt
```

UPARSE COMMANDS AND PARAMETERS FOR ITS-10 DATASET

Parameter tuning on the ITS-10-1 dataset: the parameters are chosen balancing the sequence quality and the sensitivity to low abundance species. In this case we choose a quality threshold of 3 and a length threshold of 150 allowing more then 70% of reads passing the filter (2).

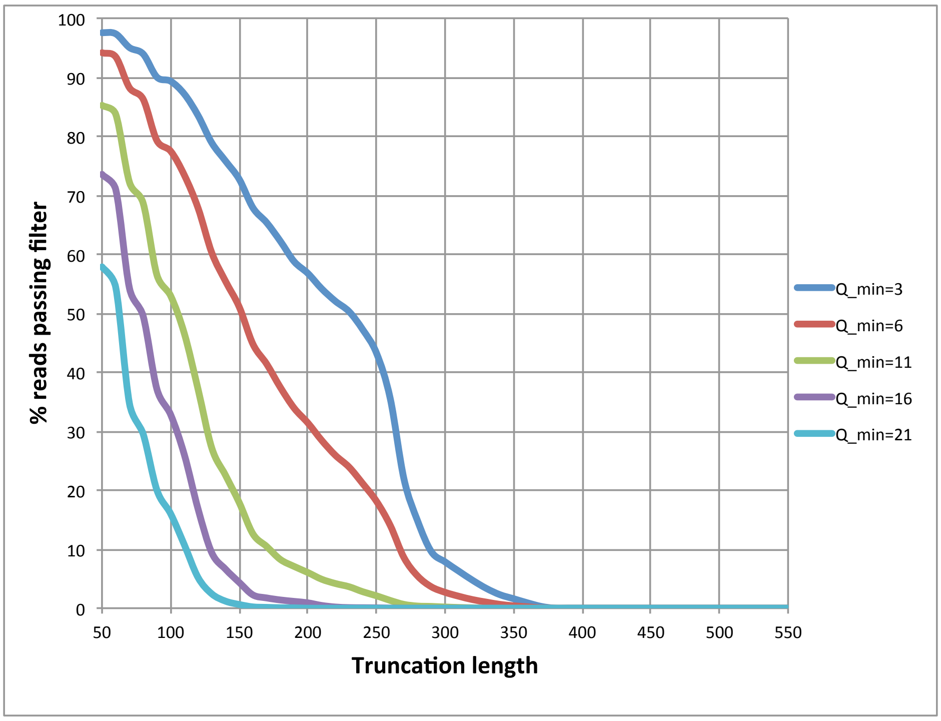

Command lines:

```
$ usearch=./usearch7.0.1090_i86osx32
# Strip barcodes and primers
$ python ./python_scripts/fastq_strip_barcode_relabel2.py seqs.fastq \
    GCATCGATGAAGAACGCAGC barcodes.fasta strip > seqs_strip.fastq
# Quality filtering and length truncation
$ $usearch -fastq_filter seqs_strip.fastq -fastaout seqs_filtered.fasta \
    -fastq_truncqual 3 -fastq_trunclen 150
# Dereplication
$ $usearch -derep_fulllength seqs_filtered.fasta -output seqs_derep.fasta \
    -sizeout
# Discard singletons
$ $usearch -sortbysize seqs_derep.fasta -output seqs_sorted.fasta -minsize 2
# UPARSE-OTU
$ $usearch -cluster_otus seqs_sorted.fasta -otus seqs_otus_tmp.fasta
# Label OTU sequences OTU_1, OTU_2...
$ python ./python_scripts/fasta_number.py seqs_otus_tmp.fasta OTU_ > \
seqs_otus.fasta
# Map reads back to OTUs
$ $usearch -usearch_global seqs_filtered.fasta -db seqs_otus.fasta -strand \
plus -id 0.97 -uc seqs_map.uc
# Create OTU table
$ python ./python_scripts/uc2otutab.py seqs_map.uc > seqs_otu_table.txt
```

UPARSE COMMANDS AND PARAMETERS FOR THE HMP DATASET

The UPARSE parameters for the HMP dataset are the same used in (2) (Supplementary Material) .

```
$ usearch=./usearch7.0.1090_i86osx32
# Strip barcodes and primers
# V1-V3: ATTACCGCGGCTGCTGG
# V3-V5: CCGTCAATTCMTTTRAGT
# V6-V9: TACGGYTACCTTGTTAYGACTT
$ python ./python_scripts/fastq_strip_barcode_relabel2.py seqs.fastq \
    ATTACCGCGGCTGCTGG barcodes.fasta strip > seqs_strip.fastq
# Quality filtering and length truncation
$ $usearch -fastq_filter seqs_strip.fastq -fastaout seqs_filtered.fasta \
    -fastq_truncqual 16 -fastq_trunclen 250
# Dereplication
$ $usearch -derep_fulllength seqs_filtered.fasta -output seqs_derep.fasta \
    -sizeout
# Discard singletons
$ $usearch -sortbysize seqs_derep.fasta -output seqs_sorted.fasta -minsize 2
# UPARSE-OTU
$ $usearch -cluster_otus seqs_sorted.fasta -otus seqs_otus_tmp.fasta
# Label OTU sequences OTU_1, OTU_2...
$ python ./python_scripts/fasta_number.py seqs_otus_tmp.fasta OTU_ > \
seqs_otus.fasta
# Map reads back to OTUs
$ $usearch -usearch_global seqs_filtered.fasta -db seqs_otus.fasta -strand \
plus -id 0.97 -uc seqs_map.uc
# Create OTU table
$ python ./python_scripts/uc2otutab.py seqs_map.uc > seqs_otu_table.txt
```

QIIME COMMANDS AND PARAMETERS FOR 16S-R AND 16S-10 DATASETS

The QIIME pipeline is build using the information taken from http://qiime.org/tutorials/tutorial.html. Length and quality thresholds were the same used for MICCA (length threshold=300 and quality threshold=24).

```
$ split_libraries.py -m map.txt -f seqs.fna -q seqs.qual -z truncate_only \
    -o split -l 300 -s 24 -b 8
$ pick_otus.py -i split/seqs.fna -o split/otus
$ pick_rep_set.py -i split/otus/seqs_otus.txt -f split/seqs.fna \
    -o split/otus/rep.fna
$ make_otu_table.py -i split/otus/seqs_otus.txt -o split/otus/otu_table.biom
$ biom convert -i split/otus/otu_table.biom -o split/otus/otu_table.txt –b
```

Singletons are discarded from the OTU table using a custom script.

QIIME COMMANDS AND PARAMETERS FOR ITS-10 DATASETS

The QIIME pipeline is build using the information taken from http://qiime.org/tutorials/tutorial.html. Length and quality thresholds were the same used for MICCA (length threshold=250 and quality threshold=20).

```
$ split_libraries.py -m map.txt -f seqs.fna -q seqs.qual -z truncate_only \
    -o split -l 250 -s 20 -b 8
$ pick_otus.py -i split/seqs.fna -o split/otus
$ pick_rep_set.py -i split/otus/seqs_otus.txt -f split/seqs.fna \
    -o split/otus/rep.fna
$ make_otu_table.py -i split/otus/seqs_otus.txt -o split/otus/otu_table.biom
$ biom convert -i split/otus/otu_table.biom -o split/otus/otu_table.txt –b
```

Singletons are discarded from the OTU table using a custom script.

QIIME COMMANDS AND PARAMETERS FOR THE HMP DATASETS

The QIIME pipeline is build using the information taken from http://qiime.org/tutorials/tutorial.html. Length and quality thresholds were the same used for MICCA (length threshold=300 and quality threshold=20)

```
$ split_libraries.py -m map.txt -f seqs.fna -q seqs.qual -z truncate_only \
    -o split -l 300 -s 20 -b 11
$ pick_otus.py -i split/seqs.fna -o split/otus
$ pick_rep_set.py -i split/otus/seqs_otus.txt -f split/seqs.fna \
    -o split/otus/rep.fna
$ make_otu_table.py -i split/otus/seqs_otus.txt -o split/otus/otu_table.biom
$ biom convert -i split/otus/otu_table.biom -o split/otus/otu_table.txt –b
```

MOTHUR COMMANDS AND PARAMETERS FOR 16S-R DATASETS

The 16S-R dataset was analyzed following the recommended procedure at http://www.mothur.org/wiki/Schloss_SOP and in (2).

```
fastq.info(fastq=seqs_reads.fastq)

trim.seqs(fasta=seqs_reads.fasta, oligos=oligos, qfile=seqs _reads.qual,
maxambig=0, maxhomop=8, flip=F, bdiffs=1, pdiffs=2, qwindowaverage=24,
qwindowsize=50, minlength=300)

unique.seqs(fasta=seqs _reads.trim.fasta)

align.seqs(fasta=seqs_reads.trim.unique.fasta, reference=silva.bacteria.fasta)

screen.seqs(fasta=seqs_reads.trim.unique.align, name=seqs_reads.trim.names,
end=22534, optimize=start, criteria=95)

filter.seqs(fasta=seqs_reads.trim.unique.good.align, vertical=T, trump=.)

unique.seqs(fasta=seqs_reads.trim.unique.good.filter.fasta,
name=seqs_reads.trim.names)

pre.cluster(fasta=seqs_reads.trim.unique.good.filter.unique.fasta, name= seqs
_reads.trim.unique.good.filter.names, diffs=2)

chimera.uchime(fasta=seqs_reads.trim.unique.good.filter.unique.precluster.fasta,
name=seqs_reads.trim.unique.good.filter.unique.precluster.names)

remove.seqs(accnos=seqs_reads.trim.unique.good.filter.unique.precluster.accnos,
fasta=seqs_reads.trim.unique.good.filter.unique.precluster.fasta,
name=seqs_reads.trim.unique.good.filter.unique.precluster.names)

dist.seqs(fasta=seqs_reads.trim.unique.good.filter.unique.precluster.fasta,
cutoff=0.15)

cluster(column=seqs_reads.trim.unique.good.filter.unique.precluster.dist,
name=seqs_reads.trim.unique.good.filter.unique.precluster.names)
```

RDP CLASSIFIER (v 2.8) COMMAND LINE FOR THE HMP DATASETS

```
$ java -Xmx2g -jar rdp_classifier.jar classify -c 0 -f fixrank -g 16srrna -o
taxa_rdp.txt representatives.fasta
```

REFERENCES

1. Dethlefsen, L. and Relman, D.A. (2011) Incomplete recovery and individualized responses of the human distal gut microbiota to repeated antibiotic perturbation. Proc Natl Acad Sci U S A, 108 Suppl 1, 4554-4561.

2. Edgar, R.C. (2013) UPARSE: highly accurate OTU sequences from microbial amplicon reads. Nat Methods, 10, 996-998.
